# Association between Plasma Fibrinogen Levels and Mortality in Acute-on-Chronic Hepatitis B Liver Failure

**DOI:** 10.1155/2015/468596

**Published:** 2015-04-16

**Authors:** Zhexin Shao, Ying Zhao, Limin Feng, Guofang Feng, Juanwen Zhang, Jie Zhang

**Affiliations:** ^1^Department of Hospital Management Office, The First Affiliated Hospital, College of Medicine, Zhejiang University, Qingchun Road No. 79, Hangzhou 310003, China; ^2^Department of Clinical Laboratory, The First Affiliated Hospital, College of Medicine, Zhejiang University, Qingchun Road No. 79, Hangzhou 310003, China; ^3^Women's Hospital, School of Medicine, Zhejiang University, Xueshi Road No. 1, Hangzhou 310006, China

## Abstract

Acute-on-chronic liver failure (AoCLF) is the most common type of liver failure and is associated with high mortality. Fibrinogen is critical in maintaining primary and secondary hemostasis. Therefore, we prospectively analyzed the association between fibrinogen and outcomes in AoCLF patients. Plasma fibrinogen was measured in 169 AoCLF, 173 chronic hepatitis B (CHB), and 171 healthy patients using a coagulation method. The predictive ability of fibrinogen for 3-month mortality in AoCLF patients was assessed using receiver operating characteristic (ROC) curve and multivariable logistic regression analyses. Plasma fibrinogen was significantly lower in nonsurvivor AoCLF patients compared with survivor AoCLF, CHB, and control patients. The sensitivity, specificity, and area under the ROC curve of 1/fibrinogen predicting mortality in AoCLF patients were 66.7%, 72.5%, and 0.746 (95% confidence interval (CI): 0.672–0.820, *P* < 0.001), and the fibrinogen cutoff value was 0.90 g/L. On multivariate logistic regression analysis, low fibrinogen was an independent factor predicting mortality (odds ratio: 0.304; 95% CI: 0.094–0.983; *P* = 0.047). Nonsurvivor AoCLF patients had significantly decreased fibrinogen levels, suggesting that low plasma fibrinogen may be a useful predictor of poor prognosis in AoCLF patients.

## 1. Introduction

Hepatitis B virus (HBV) infection is a global health threat with approximately 1% of HBV-infected patients developing liver failure, a prevalence that primarily reflects the HBV virulence combined with spontaneous or induced factors [1]. In China, HBV-related acute-on-chronic liver failure (AoCLF) is the most common type of liver failure and is caused by an acute severe exacerbation of chronic hepatitis B (CHB); as a result, AoCLF is associated with high mortality [2, 3]. Although the pathophysiology of AoCLF in CHB remains poorly understood, the clinical and immunological features of AoCLF in CHB are distinctive from those in other causes of AoCLF, such as drug use and alcohol abuse [1].

The liver plays a key role in hemostasis regulation [4]. The liver parenchymal cells produce most of the factors and inhibitors in the clotting and fibrinolytic systems, and the liver greatly aids in clearing activated clotting or fibrinolysis enzymes from the circulation, which protect against both hemorrhage and inappropriate activation of coagulation [4, 5]. In patients with liver disease, hemostasis tests may be required to evaluate the severity of hepatocellular failure [6]. Fibrinogen (coagulation factor I) is a 300 kDa soluble plasma glycoprotein comprising two identical subunits, each containing three polypeptide chains (A*α*, B*β*, and *γ*) and synthesized in the liver [7]. Fibrinogen plays several key roles in maintaining both primary and secondary hemostasis and is converted by thrombin into an insoluble fibrin network that, together with platelet aggregates, induces hemostasis in response to rupture of the endothelium [8]. Cong et al. observed that the plasma fibrinogen concentration in liver cirrhosis patients progressively decreased as liver cirrhosis worsened, indicating a close relationship between the severity of cirrhosis and hemostatic changes [9]. Zhou et al. found that the plasma concentration and function of fibrinogen persisted in a low coagulation state in patients with hepatic-nephrotic disease [10]. However, the fibrinogen function in patients with AoCLF has not been previously reported. Therefore, we prospectively analyzed the association between fibrinogen and clinical outcomes in AoCLF patients.

## 2. Subjects and Methods

### 2.1. Subjects

A total 169 patients with AoCLF and 173 patients with CHB, admitted to the Department of Infectious Disease, The First Affiliated Hospital, College of Medicine, Zhejiang University, China, between January 2012 and December 2013, were recruited. AoCLF is defined as an acute hepatic insult manifesting as jaundice and coagulopathy and complicated by ascites and/or encephalopathy within 4 weeks in a patient with previously diagnosed or undiagnosed chronic liver disease [11]. AoCLF often develops subsequent to acute decompensation of cirrhosis, and complications include ascites, encephalopathy, upper gastrointestinal hemorrhage, renal dysfunction, and bacterial infections [12, 13]. In the present 169 AoCLF patients, 88 patients had cirrhosis with decompensation. AoCLF was accompanied by ascites in 79 patients, encephalopathy in 56, upper gastrointestinal hemorrhage in 16, renal dysfunction in 20, and bacterial infections in 46 patients. Forty-five patients exhibited more than two complications. CHB, excluding liver cirrhosis, was defined as previous hepatitis B or hepatitis B surface antigen (HBsAg) positivity for >6 months and persistently positive HBsAg and/or HBV DNA [14]. Exclusion criteria included age less than 18 years; concurrent hepatitis C virus, hepatitis D virus, hepatitis G virus, and/or human immunodeficiency virus infection; any autoimmune liver disease; hepatocellular carcinoma; metabolic liver disease; alcoholic liver disease; liver transplantation; pregnancy; and serious disease in other organ systems. A total of 171 healthy control patients who were HBsAg negative with normal liver function, renal function, and blood lipid levels measured at an annual health examination at the healthcare center of The First Affiliated Hospital of Zhejiang University were also recruited.

### 2.2. Ethics Statement

This study was approved by the Ethics Committee of The First Affiliated Hospital of the Medical College at Zhejiang University in China and was performed in accordance with the Helsinki Declaration. All participants provided written informed consent.

### 2.3. Specimen Collection

All specimens for blood indicators and coagulation measurements were collected by venipuncture into 5 mL drying VACUETTE vacutainers (Greiner Bio-One GmbH, Kremsmunster, Austria) and 5 mL Becton Dickinson vacutainers containing 0.5 mL 0.109 M sodium citrate (Becton Dickinson, Franklin Lakes, NJ, USA) in the morning after a 12-hour fast. The samples were sent to the laboratory, and the serum and plasma were isolated by centrifugation (10 min, 3000 g).

### 2.4. Laboratory Techniques

The alanine aminotransferase (ALT), total bilirubin (TBIL), albumin (ALB), and creatinine (Cr) were measured with a Hitachi 7600 automatic biochemical analyzer (Hitachi Ltd., Tokyo, Japan) using Roche Diagnostics GmbH reagents (Roche Diagnostics, GmbH, Mannheim, Germany). The fibrinogen and prothrombin time (PT) were measured by the coagulation method using a Sysmex CA7000 System (Sysmex, Kobe, Japan) with SIEMENS reagents (SIEMENS, Marburg, Germany). The D-dimer was measured by turbidimetric method also using a Sysmex CA7000 System with SIEMENS reagents. A real-time fluorescent PCR system (7300; Applied Biosystems, Inc., Carlsbad, CA, USA) was used to measure the HBV DNA concentration with a lower limit of detection of 12 IU/mL. HBsAg and HBeAg were measured on an Architect Ci8200 automated biochemistry analyzer (Abbott Laboratories, Abbott Park, IL, USA) using Abbott reagents. The severity of liver disease was quantified using the model for end-stage liver disease (MELD) score, which is calculated from the serum TBIL, creatinine, and international normalized ratio (INR) of the prothrombin time [15].

### 2.5. Statistical Analysis

Statistical analysis was performed using SPSS16.0 (SPSS Inc., IL, USA). Continuous data were presented as the mean ± standard deviation (SD) or median (range) and categorical data as percentages, as appropriate. For continuous variables, the differences between two groups were assessed with the independent samples *t*-test or the Mann-Whitney *U* test, as appropriate. Multiple comparisons were performed by one-way analysis of variance (ANOVA) or the Kruskal-Wallis *H* test. Categorical variables were analyzed using the Chi-square test. Spearman's rank correlation test was used for the correlation analysis. After reciprocal transformation of the fibrinogen concentration (1/fibrinogen), a receiver operating characteristic (ROC) curve was generated, and the area under the curve (AUC) was calculated to identify the cutoff value of fibrinogen to predict mortality in patients with AoCLF. Univariate and multivariable stepwise logistic regression were used to evaluate independent clinical parameters predicting mortality. Statistical significance was defined at *P* < 0.05 (two-sided).

## 3. Results

### 3.1. Decreased Plasma Fibrinogen Level in Patients with AoCLF

**Figure 1:**
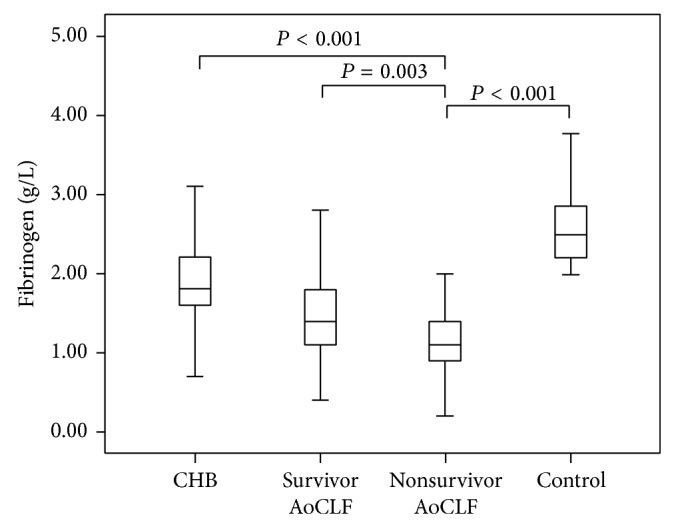
Plasma fibrinogen levels in survivor AoCLF, nonsurvivor AoCLF, CHB, and control groups. AoCLF, acute-on-chronic hepatitis B liver failure; CHB, chronic hepatitis B.

A total of 173 patients with CHB, 169 patients with AoCLF, and 171 healthy control patients were enrolled in our study. The clinical characteristics of the patients are listed in Table 1. The ALT, TBIL, Alb, Cr, PT, fibrinogen, D-dimer, and INR differed significantly among the three groups (*P* < 0.05). The MELD score, decompensation, and mortality also differed significantly between the CHB and AoCLF groups (*P* < 0.05). The fibrinogen level was 1.39 ± 0.58 g/L in the AoCLF group, which was significantly lower than the levels in the healthy control and CHB groups. The fibrinogen concentration in nonsurvivor AoCLF patients was lower than that in survivor AoCLF patients (1.22 ± 0.62 g/L versus 1.48 ± 0.54 g/L, resp., *P* = 0.003, Figure 1).

### 3.2. Correlation Analysis between Fibrinogen and Other Variables

Spearman's correlation analysis was employed to determine the correlation between the fibrinogen concentration and other variables. The fibrinogen concentration in the AoCLF patients correlated with age, PT, INR, ALB, TBIL, D-dimer, and MELD (*P* < 0.05; *r* = −0.184, −0.685, −0.680, 0.163, −0.322, −0.357, and −0.472, resp.), in CHB patients correlated with PT, INR, ALB, ALT, TBIL, and MELD (*P* < 0.05; *r* = −0.446, −0.473, 0.338, −0.181, −0.350, and −0.380, resp.), and in the control group correlated with age, Cr, and TBIL (*P* < 0.05; *r* = 0.205, −0.204, and −0.260, resp.). The PT and INR both had a Spearman correlation coefficient (*r*-value) greater than 0.5 with the fibrinogen level in AoCLF patients.

### 3.3. Clinical Characteristics of AoCLF Patients Based on Plasma Fibrinogen Levels

To explore the association of fibrinogen with AoCLF and mortality, the 169 AoCLF patients were divided into three groups according to the fibrinogen tertiles (group 1: fibrinogen < 1.10 g/L; group 2: fibrinogen 1.10–1.50 g/L; and group 3: fibrinogen > 1.50 g/L). The clinical characteristics of the AoCLF patients according to the fibrinogen group are listed in Table 2. The gender, age, TBIL, PT, INR, D-dimer, MELD score, decompensation, and mortality differed significantly between the three groups (*P* < 0.05). Patients with the lowest fibrinogen level (group 1) had the highest TBIL, PT, INR, D-dimer, MELD score, decompensation, and mortality compared with groups 2 and 3.

### 3.4. Association of Fibrinogen and 3-Month Mortality of AoCLF Patients

The 3-month mortality rate decreased as the fibrinogen level increased at 53.8% (28/52) in group 1, 32.3% (20/62) in group 2, and 21.8% (12/55) in group 3 (Table 2). ROC curve analysis was applied to estimate the fibrinogen level and MELD score predicting the mortality of AoCLF patients. The sensitivity, specificity, and AUC of 1/fibrinogen were 66.7%, 72.5%, and 0.746, respectively (95% confidence interval (CI): 0.672–0.820; *P* < 0.001), and the cutoff fibrinogen level was 0.90 g/L. The cutoff value, sensitivity, specificity, and AUC of the MELD score were 21.72, 80.0%, 84.4%, and 0.890, respectively (95% CI: 0.836–0.944; *P* < 0.001).

Fibrinogen, TBIL, and MELD score were associated with the mortality of AoCLF patients (*P* = 0.033, *P* < 0.001, and *P* = 0.028, resp.) by stepwise regression selected from the age, gender, fibrinogen, Cr, ALT, ALB, TBIL, PT, INR, D-dimer, and MELD score. Further multivariate logistic regression analysis after adjusting for the TBIL and MELD score showed that the fibrinogen level, TBIL, and MELD score were independent factors predicting mortality with odds ratios (OR) as follows: 0.304 (95% CI: 0.094–0.983, *P* = 0.047); 1.008 (95% CI: 1.005–1.012, *P* < 0.001); and 1.090 (95% CI: 1.006–1.181, *P* = 0.035), respectively.

## 4. Discussion

Fibrinogen provides the supporting framework in hemostasis and coagulation [16, 17]. The major physiological function of fibrinogen is to form the fibrin that binds platelets and certain plasma proteins into a hemostatic plug, forming bridges responsible for platelet aggregation. The fibrin matrix is also essential in the regulation of fibrinolysis and facilitation of cell attachment in wound healing [16]. During the first phase of clot formation, fibrinogen serves as a molecular link mediating platelet aggregation, and during the second phase, fibrinogen initiates fibrin polymerization and converts to a fibrin matrix that gives the clot shape, strength, flexibility, and stability [16–18]. Fibrinogen is an old index routinely used to assess pathological alterations of the hemostatic and coagulation systems [16]. Recently, new accumulating data indicate that fibrinogen plays a critical role in achieving and maintaining hemostasis, particularly in patients with an acquired fibrinogen deficiency [19]. Acquired fibrinogen deficiency may emerge secondary to some conditions that reduce fibrinogen levels and/or render fibrinogen dysfunctional [20]. Liver disease can cause acquired fibrinogen deficiency and may require substitution therapy with fibrinogen concentrate [19, 21, 22].

Hemostasis is intimately related to liver function because the liver synthesizes most of the coagulation proteins and inhibitors such as fibrinogen and factors II, V, VII, IX, XI, XII, and XIII. The reticuloendothelial system of the liver also serves an important role in the clearance of activation products [23]. The extent of coagulation abnormalities depends on the severity of liver dysfunction, and the clinical manifestations of liver disease depend on the severity of hemostatic impairment [5, 23]. Severe coagulopathy and hemorrhage risk in liver disease are more frequently observed in acute liver failure, chronic liver failure, and liver cirrhosis which is due to the decreased production of blood pro- and anticoagulation proteins in the liver [24]. Severe acute hepatitis, liver failure, and end-stage liver disease are associated with marked abnormalities in screening tests, including markedly elevated PT/APTT and very low fibrinogen levels. Moreover, the fibrinogen produced in such patients has an increased sialic acid component, which renders the fibrinogen molecule abnormal and prolongs thrombin time [5, 23, 24].

In our study, the AoCLF patients had significantly prolonged PT/INR and lower fibrinogen levels compared with the CHB patients and control group, and nonsurvivor AoCLF patients had the significantly lowest fibrinogen level compared with the survivor AoCLF patients, CHB patients, and control group. This outcome may reflect the hepatic synthetic capacity, which was worst in the nonsurvivor AoCLF patients, and the decreased fibrinogen level, which was significantly associated with liver damage. Several studies have also reported that fibrinogen and fibrin levels were decreased in certain liver diseases [25–27]. In one study, Mammen [25] reported that fibrinogen and fibrin abnormalities were associated with various liver diseases after several types of liver disturbance. Fibrinogen levels were decreased in patients with moderate and severe hepatocellular damage (except surgery), but fibrin(ogen) split products (FSPs) were increased, while in patients with mild hepatocellular damage, the fibrinogen levels and FSPs were normal [25]. Soria et al. found that fibrinogen levels were usually decreased, especially in severe forms of cirrhosis, and abnormal fibrinogen molecules could be detected [26]. Increased circulating soluble fibrin complexes (sFC) may indicate conversion of fibrinogen to fibrin by thrombin [28]. A study by Kim et al. observed that sFC was the biomarker most strongly associated with disseminated intravascular coagulation in liver cirrhosis patients compared with other markers [27].

The prognosis of AoCLF in CHB is extremely poor, and the 3-month mortality rate without liver transplantation is reportedly greater than 50% [29]. Our results also demonstrated the highest mortality, decompensation, MELD score, TB, D-dimer, and PT/INR in AoCLF patients in the lowest fibrinogen group (group 1) compared with the values in groups 2 and 3. Alternatively, 1/fibrinogen can be used to predict the mortality of AoCLF patients by ROC curve analysis. Although the prediction power was lower than that of the MELD score, fibrinogen as a single index was easier and more convenient than the MELD score. Moreover, logistic regression analyses suggested that low fibrinogen level may serve as an independent predictor for mortality in AoCLF patients; we observed that a lower fibrinogen was associated with higher mortality and decompensation. Shi et al. found that AoCLF patients with decompensation had significantly higher long-term mortality rates (post-28-days) [13]. Notably, multiple Asian studies identified several factors associated with adverse outcomes, including preexisting cirrhosis, prolonged PT, elevated bilirubin, elevated serum ferritin, low albumin, low platelet count, and the presence of encephalopathy or ascites [30–32]. Our study complements their studies and indicates that low fibrinogen also can be used to predict the prognosis in AoCLF patients.

In conclusion, we evaluated the association between fibrinogen and clinical outcomes in AoCLF patients and found abnormally low fibrinogen levels in AoCLF patients, especially nonsurvivor AoCLF patients, which suggests that low plasma fibrinogen may be a useful indicator for predicting a poor prognosis in AoCLF patients. However, our study requires confirmation by additional studies due to the small sample size and single-center methodology.

## Figures and Tables

**Table 1:** Clinical characteristics of enrolled participants.

Variables	Control (171)	CHB (173)	AoCLF (169)	*P* value
Female/male	37/134	35/138	32/137	0.825
Age (year)	46.43 ± 8.80	44.47 ± 9.82	46.08 ± 12.90	0.190
HBsAg positive	0	173	169	—
HBeAg positive	0	92	78	—
HBV DNA positive	0	173	169	—
TBIL (*μ*mol/L)	12 (6–49)	23 (5–430)	209 (10–947)^∗#^	<0.001
ALT (U/L)	17 (7–48)	65 (9–1838)	65 (10–3686)^#^	<0.001
ALB (g/L)	46.5 (40–55.1)	37.8 (24.4–52.4)	32.4 (20.4–46.2)^∗#^	<0.001
Cr (*μ*mol/L)	75 (38–100)	66 (29–154)	65 (23–760)	0.004
PT (S)	10.8 (9.4–12.7)	13.0 (10.1–24.1)	22.2 (11.1–120)^∗#^	<0.001
Fibrinogen (g/L)	2.58 ± 0.49	1.88 ± 0.56	1.39 ± 0.58^#∗^	<0.001
INR	0.94 (0.82–1.10)	1.14 (0.88–2.05)	1.92 (0.97–10.4)^∗#^	<0.001
D-dimer (*μ*g/L FEU)	150 (2–510)	337 (30–5550)	1892 (150–43858)^∗#^	<0.001
MELD score		6.06 (0.17–20.29)	18.88 (3.98–57.17)	<0.001
Decompensation (yes/no)		0/173	88/81^*^	—
Mortality (yes/no)		0/173	60/109^*^	—

CHB, chronic hepatitis B; AoCLF, acute-on-chronic hepatitis B liver failure; HBsAg, hepatitis B surface antigen; HBeAg, hepatitis B e antigen; HBV, hepatitis B virus; TBIL, total bilirubin; ALT, alanine aminotransferase; ALB, albumin; Cr, creatinine; PT, prothrombin time; INR, international normalized ratio; FEU, fibrinogen equivalent units; MELD score, the model for end-stage liver disease score.

*P* value: comparison among these three groups. ^#^ *P* < 0.05 versus the control group. ^*^ *P* < 0.05 versus the CHB group.

**Table 2:** Clinical characteristics of the AoCLF patients according to the plasma fibrinogen tertiles.

Variables	Group 1 (52)	Group 2 (62)	Group 3 (55)	*P* value
Fibrinogen (g/L)	<1.10	1.10~1.50	>1.50	
Female/male	21/31^#∗^	9/53^☆^	2/53	<0.001
Age (year)	49.42 ± 12.51^*^	46.15 ± 13.26	42.84 ± 12.90	0.030
TBIL (*μ*mol/L)	352 (40–826)^#∗^	223 (42–947)	117 (10–804)	<0.001
ALT (U/L)	81 (10–3686)	61 (15–2401)	55 (20–1500)	0.299
ALB (g/L)	31.6 (20.4–46.2)	32.3 (24.3–44.7)	32.8 (23.4–46.1)	0.112
Cr (*μ*mol/L)	65 (24–263)	66 (23–760)	66 (28–273)	0.650
PT (S)	28.2 (14.2–120)^#∗^	22.8 (14.1–43.1)^☆^	17.2 (11.1–29.7)	<0.001
INR	2.41 (1.23–10.40)^#∗^	1.96 (1.22–3.60)^☆^	1.49 (0.97–2.51)	<0.001
D-dimer (*μ*g/L FEU)	3090 (380–6990)^#∗^	1715 (150–43858)	1070 (150–7080)	<0.001
MELD score	24.58 (11.27–57.17)^#∗^	19.56 (5.2–50.07)^☆^	15.74 (3.98–39.04)	<0.001
Decompensation (yes/no)	35/17^#∗^	30/32	23/32	0.024
Mortality (yes/no)	28/24^#∗^	20/42	12/43	0.002

AoCLF, acute-on-chronic hepatitis B liver failure; TBIL, total bilirubin; ALT, alanine aminotransferase; ALB, albumin; Cr, creatinine; PT, prothrombin time; INR, international normalized ratio; MELD score, the model for end-stage liver disease score.

*P* value: comparison among the three groups. ^#^ *P* < 0.05, group 1 versus group 2. ^*^ *P* < 0.05, group 1 versus group 3. ^☆^ *P* < 0.05, group 2 versus group 3.
